# Serial selection for invasiveness increases expression of miR-143/miR-145 in glioblastoma cell lines

**DOI:** 10.1186/1471-2407-12-143

**Published:** 2012-04-10

**Authors:** Sunwoo Koo, Gail S Martin, Kevin J Schulz, Matthew Ronck, L Gerard Toussaint

**Affiliations:** 1 Department of Neuroscience and Experimental Therapeutics, Texas A&M Health Science Center College of Medicine, Bryan, TX 77843, USA; 2 The Texas Brain and Spine Institute, Bryan, TX 77807, USA

**Keywords:** Glioblastoma, MicroRNA-143, MicroRNA-145, Invasion

## Abstract

**Background:**

Glioblastoma multiforme (GBM) is the most common primary central nervous system malignancy and its unique invasiveness renders it difficult to treat. This invasive phenotype, like other cellular processes, may be controlled in part by microRNAs - a class of small non-coding RNAs that act by altering the expression of targeted messenger RNAs. In this report, we demonstrate a straightforward method for creating invasive subpopulations of glioblastoma cells (IM3 cells). To understand the correlation between the expression of miRNAs and the invasion, we fully profiled 1263 miRNAs on six different cell lines and two miRNAs, miR-143 and miR-145, were selected for validation of their biological properties contributing to invasion. Further, we investigated an ensemble effect of both miR-143 and miR-145 in promoting invasion.

**Methods:**

By repeated serial invasion through Matrigel^®^-coated membranes, we isolated highly invasive subpopulations of glioma cell lines. Phenotypic characterization of these cells included *in vitro *assays for proliferation, attachment, and invasion. Micro-RNA expression was compared using miRCURY arrays (Exiqon). In situ hybridization allowed visualization of the regional expression of miR-143 and miR-145 in tumor samples, and antisense probes were used investigate *in vitro *phenotypic changes seen with knockdown in their expression.

**Results:**

The phenotype we created in these selected cells proved stable over multiple passages, and their microRNA expression profiles were measurably different. We found that two specific microRNAs expressed from the same genetic locus, miR-143 and miR-145, were over-expressed in our invasive subpopulations. Further, we also found that combinatorial treatment of these cells with both antisense-miRNAs (antimiR-143 and -145) will abrogated their invasion without decreasing cell attachment or proliferation.

**Conclusions:**

To best of our knowledge, these data demonstrate for the first time that miR-143 and miR-145 regulate the invasion of glioblastoma and that miR-143 and -145 could be potential therapeutic target for anti-invasion therapies of glioblastoma patients.

## Background

Glioblastoma multiforme (GBM) is the most common primary brain tumor in adults, and the discovery of this tumor in patients portends a dismal prognosis. The median survival of only 12-18 months is due, at least in part, to its invasive phenotype - rendering complete surgical resection nearly impossible. Even more distressing to patients, family members, and caregivers is the loss of neurological function that accompanies tumor invasion, recurrence, and repeated treatments. Understanding and controlling the invasive phenotype of glioblastoma offers hope of improving therapies and preserving meaningful function.

Currently, various investigators are completing, or have recently finished, clinical trials of small molecule inhibitors in glioblastoma patients based on molecular observations of protein expression and signaling cascades (e.g. inhibitors of VEGF, TGF-beta, EGFR, m-TOR) [1]. A new molecular signaling paradigm has been described in the last decade, offering more potential therapeutic targets to alter the malignant phenotype of this disease.

MicroRNAs (miRNAs) are noncoding small RNA molecules which regulate post-transcriptional gene expression and have been proposed as novel cancer biomarkers and potential targets of new anticancer therapies [2]. Several groups have reported data describing the microRNA expression profiles of glioblastma [3,4]. For example, miR-124a, -125a, -29b, -7, -128 have been reported as a glioblastma tumor suppressors while miR-21 increases glioblastoma cell growth by targeting p53 and TGF-β [4,5]. In recent years, a handful of microRNA species have been linked specifically to glioblastoma brain invasion [5-7].

Herein, we describe a simple and reproducible method for creating subpopulations of glioblastoma cells with enhanced invasive properties. We present microRNA expression data differentiating these invasive cells, and provide a rationale for investigating miR-145 and mir-143 further. Finally, we confirm the expression of miR-143 and miR-145 in invasive locations within glioblastoma samples and, via knockdown experiments, illustrate reduced invasion when their expression is abrogated.

## Methods

### Cell lines and culture conditions

The human glioma cell lines U87MG, U251, U373 and the rat glioma cell line C6 were obtained from the American Type Culture Collection (ATCC, Manassas, VA, USA). The cells were grown in Dulbecco's modified Eagle's medium (DMEM, Cellgro Media tech)) supplemented with 10% heat-inactivated fetal bovine serum, penicillin (10 IU/ml), and streptomycin (10 ug/ml). The cells were maintained at 37°C in a humidified air atmosphere at 5% CO2.

### Serial selection for a sub-population of invasive cells using Boyden chambers

For selection of invasive cells, a suspension of 300,000 tumor cells/mL in serum-free DMEM (500 ul total volume) was plated in the upper chamber of a Boyden-type manifold, over a Matrigel-coated membrane (24-well insert; pore size, 8 um; BD Biosciences). DMEM medium containing 10% FBS in the lower chamber served as the chemoattractant. After incubation for 21 h at 37°C, those non-invading cells from the upper surface of the membrane were scrubbed off with cotton swabs. The cells that invaded to the bottom surface of the insert were trypsinized and seeded into flasks containing DMEM supplemented with 10% FBS. These cells were propagated and went through the above selection procedure twice more. The cells that were collected after the third selection procedure were propagated resulting in the invasive sub-population cell line termed IM3.

### Boyden chamber invasion assay

For migration assays, a suspension of 300,000 tumor cells/ml in serum-free DMEM (500 ul total volume) was plated in the upper chamber of a Matrigel-coated membrane insert (24-well insert; pore size, 8 um; BD Biosciences). DMEM medium containing 10% FBS in the lower chamber served as the chemoattractant. The cells were incubated at 37°C for various times depending on our laboratory experience regarding their relative speeds of invasion (18 hrs for U87 and U251; 36 hrs for U373; 17 hrs for C6). The non-invading cells were removed with cotton swabs. Those cells that had migrated to the lower side of the membrane were fixed and stained with hematoxylin. The invading cells on quadruplicate membranes were quantitated by counting across a diameter of each memebrane under 40X microscopic magnification.

### Cell attachment assay

For the cell attachment assay, wells of a 24-well tissue culture plate were coated with Matrigel Basement Membrane Matrix (BD Biosciences) diluted 1:10 with serum-free medium for 2 h at room temperature. The unbound material was aspirated and the wells were rinsed with serum-free medium. Cells were detached with trypsin/EDTA and rinsed in serum free medium. Approximately 20,000 cells/ml in 10% DMEM were plated into the wells, and the plate was incubated at 37°C for 4 h. Wells were gently washed with PBS and the attached cells were fixed in 4% paraformaldehyde and stained with hematoxylin QS (Vector Laboratories) for 2 min. Wells were washed again in PBS and the stained cells were counted.

### Proliferation assay

For each cell line, parental and IM3 cells were plated at a density of 5000 cells per well, each in 8 wells of 96-well plates containing 10% FBS/DMEM and maintained at 37°C. The rate of cell proliferation was determined using CellTiter 96 AQ_ueous _One Solution (Promega, Madison, WI) at 4, 24, 48, 72 and 96 hours after plating. An MTS assay was performed by following the protocol previously reported [8]. Eight media only containing wells were used to normalize the absorbance values of the wells containing cells. Growth curves were generated with normalized absorbance compared to the 4-hour data, at 24, 36, 48, and 72 hours.

### RNA isolation

Total cellular RNA was extracted using the *mir*Vana miRNA Isolation kit (Ambion) per manufacturer's instructions for total RNA isolation. The RNA concentration was determined by spectrophotometric absorbance at 260 nm, and the quality of the RNA was determined by Agilent 2100 Bioanlayzer instrument (Agilent Technologies, Santa Clara, CA). All RNA samples showed high quality (RNA integrity number (RIN) > 9.0) and were without RNA degradation or DNA contamination.

### Real-time quantitative RT-PCR analysis

qRT-PCR was performed in triplicate by using LNA™ PCR primer sets (miR-143, -145 and U6), SYBR^®^Green PCR kit, and the ViiA™ 7 real-time PCR system (Exiqon). Total RNA extracted from U87, U251, U373, and their corresponding invasive subpopulations, was used for cDNA synthesis (Exiqon). The PCR reaction was started with cDNA (140 ng/μl) in 25 μl (total) assay mix. The PCR cycle settings were those recommended by Exiqon: 95°C, 10 min; 40 amplification cycle at 95°C (10 s) and 65°C (1 min), 1.6°C/s ramp-rate.

### Knockdown miRNA transfection

Parental and IM-3 cells from each of three cell lines (U87, U251, U373) cells were transfected with either fluorescein-labeled locked nucleic acid (LNA) antagomirs targeting miR-143 and miR-145 (Exiqon, Vedbaek, Denmark) at a final concentration of 50 nM (each probe) using Lipofectamine 2000 per manufacturer's instructions (Invitrogen, Carlsbad, CA). Control cells were transfected with an un-targeted antagomir at a final concentration of 100 nM. Transfection medium was changed 8 hours after transfection and replaced with Optimem medium (Invitrogen Carlsbad, CA) for overnight recovery. The efficiency of the transfection was assessed with fluorescence microscope. A Double knockdown transfection of U87 and U87 IM3 cells using fluorescein-labeled miR-143 and miR-145 knockdown probe at final concentration of 50 nM each and control LNA knockdown probe at final concentration of 100 nM was performed using Lipofectamine 2000 per manufacturer's instructions. Once again, culture medium was changed 8 hours after transfection and replaced with Optimem medium (Invitrogen Carlsbad, CA) for overnight recovery and the efficiency of the transfection was assessed with fluorescence microscopy. Antisense micro RNAs (antimir-143 and -145) for knockdown were purchased from Exiqon.

### Boyden chamber migration assay of knockdown miRNA Transfected cells

A suspension of 300,000 cells/mL in serum-free medium of parental and IM3 cells transfected with LNA antagomirs (against miR-143 and miR-145 or a control LNA antagomir) was plated in the upper chamber of a Boyden manifold over a Matrigel-coated membrane (24-well insert; pore size, 8 um; BD Biosciences). DMEM medium containing 10% FBS in the lower chamber served as the chemoattractant. The cells were incubated for 17-36 hours at 37°C. The non-invading cells were removed with cotton swabs. Those cells that had migrated to the lower side of the membrane were fixed and stained with DAPI. The invading cells of quadruplicate membranes were counted under the microscope at 40× magnification.

### *In situ *detection of miRNAs

*In situ *detection of miRNAs was performed on 8-10-um frozen tissue sections from xenografts of human GBM tumors in mouse brain. Sections were fixed using fresh ice-cold 4% paraformaldehyde for 1 h, acetylated in acetic anhydride/triethanolamine, and prehybridized in hybridization solution (50% formamide, 5X SSC, 0.5 mg/mL yeast tRNA, 1X Denhardt's solution) at 25°C below the predicted T_m _value of the LNA probe for 30 min. Probes (4 pmol; fluorescein isothiocyanate (FITC)-labeled LNA-modified oligonucleotide; Exiqon) complementary to the mature miRNA (miR-143 and miR-145) were hybridized to the sections for 2 h at 25°C lower than predicted T_m _value of the LNA probe. Post-hybridizations washes were performed in 0.5X SSC at 8°C above the hybridization temperature and the *in situ *hybridization signal was detected by incubation with horseradish peroxidase (HRP)-conjugated anti-FITC. The signal was then amplified using FITC-conjugated tyramide (Tyramide Signal Amplification Plus Kit, Fluorescein, Perkin-Elmer) according to the manufacturer's instructions. Slides were mounted in Vectashield Hard Set mounting medium containing 4',6-diamidino-2-phenylindole (DAPI) (Vectashield mounting medium with DAPI, Vector Laboratories) and analyzed with an Olympus CKX41 microscope equipped with a CCD camera and Olympus software.

### Statistical analysis

Data are presented as mean +/- standard deviation. For parental and IM-3 comparisons, the Student's *t *test (two-tailed) was used to determine statistical significance. A student's *t *test with a value of *P *< 0.05 was considered significant.

## Results

### Creation and characterization of highly invasive glioblastoma cell line subpopulations

**Figure 1:**
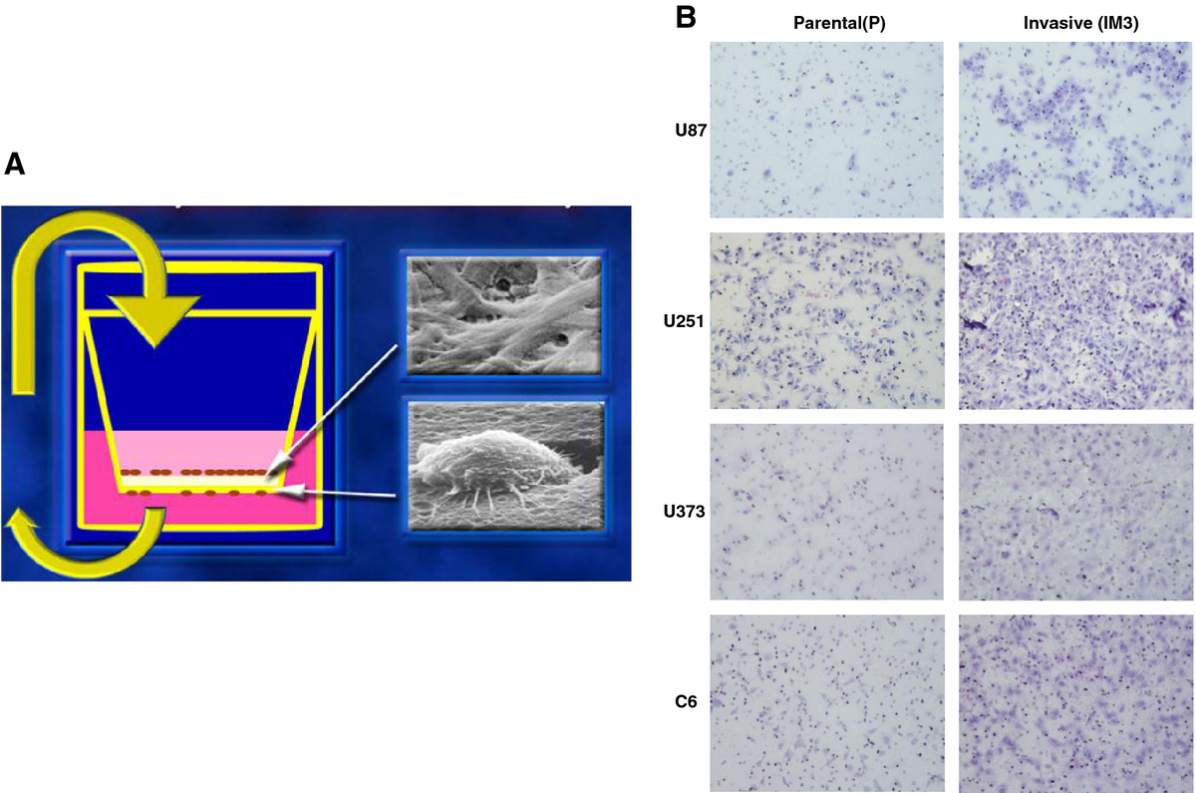
**Selection of highly invasive glioblastoma cell line subpopulations (IM3)**. (A) Schematic diagram of our serial selection algorithm (Matrigel Invasion Chamber, BD Bioscience). (B) Serial selection of cells resulted in highly invasive subpopulations of each cell type. Cells (P, parental cells) were invaded through 8 μm Matrigel^® ^pores three times for selection of invasive cells (IM3).

**Figure 2:**
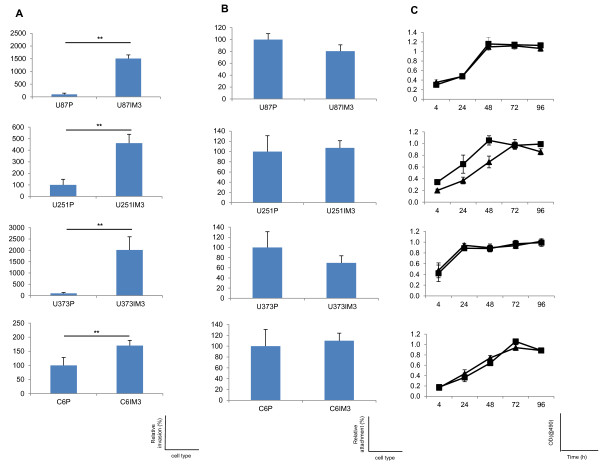
**Invasion, attachment, and proliferation assay of glioblastoma cells**. All assays were performed with four different glioblastoma cell lines: U87, U251, U373, and C6 (Top to bottom). Values in (A) and (B) are expressed as a percentage of corresponding parental line data. (A) Invasion: IM-3 subpopulations were compared to their parental lines. (B) Attachment: Cells adherent to Matrigel^® ^after 4 h were stained with hematoxylin QS and counted. (C) Proliferation: The rate of cell proliferation was evaluated with CellTiter 96 Aqueous One Solution (Promega) at five different time points, 4 h, 24 h, 48 h, 72 h and 96 h after seeding cells. Triangles - IM-3 lines, squares - parental. Data are represented as means ± SD (n = 3). P, parental cells; IM3, IM3 cells; IM3, IM3 cell line; **, p < 0.01.

Serial selection for invasion through Matrigel^®^-coated Boyden chamber membranes is a viable tool to separate glioblastoma cell lines into parental and highly-invasive sub-populations (IM3 cells). Confirmatory assays revealed 15-, 5-, 20- and 1.5-fold (U87, U251, U373 and C6 cells lines) increases in the number of invading cells when comparing selected IM3 populations to their parental counterparts (Figure 1B, 2A, Additional file 1A). This phenotypic alteration has been consistent through multiple (up to 20) passages, and at least 3 freeze-thaw cycles. Possible confounders for Boyden chamber invasion data were investigated. Enhanced attachment to Matrigel^® ^or an increase in cellular proliferation could complicate the interpretation of invasion results. We investigated both, and found no significant differences in these assays between parental and IM3 cell lines (Figure 2B, C, Additional file 1B). Serial selection resulted in a stable and predictable phenotype.

### Both miR-145 and miR-143 are expressed at a high level in IM3 cell lines

**Figure 3:**
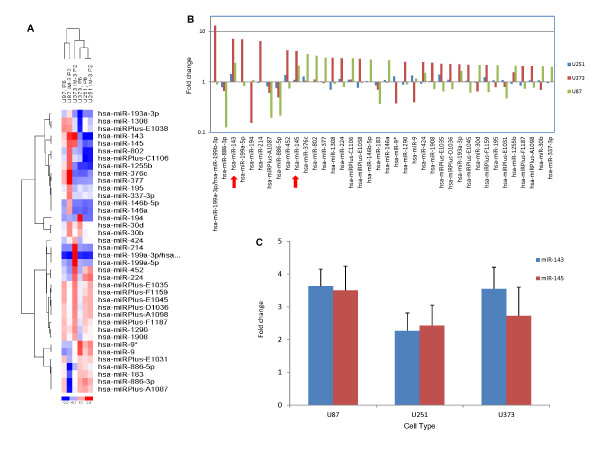
**Expression level of miR-143 and -145 in human glioblastoma cells by miRNA probe array**. The miRNA expression profile in each of eight glioblastoma cell populations was determined by using miRCURY LNA microRNA Arrays. (A) Two way hierarchical clustering of human glioblastma cells (U87, U251, U373) was represented by the heat map diagram. miRNA and sample clustering tree appear on the right and at the top respectively. The color scale at the bottom demonstrates the expression level of miRNAs across all samples: red color represents the expression level above the mean, blue color represents the expression level lower than the mean. (B) The diagram illustrates the differentially expressed miRNAs in IM3/parental cells of three human glioblastma cell lines (U87, U251, U373). Each bar represents the fold change between IM3 cells and Parental cells. Fold change > 1 indicates up-regulation of miRNA expression in IM3 cells while fold change < 1 indicates down-regulation of miRNA expression in IM3. Green: U87; Red: U373; Blue: U251 (C) The expression of miRNA was quantitated and validated with qRT-PCR analysis. All reactions were performed in triplet.

Using the human miRCURY LNA microarray platform from Exiqon, we analyzed expression of all miRBASE v.16 human microRNAs, and compared data between parental and IM3 cells (Figure 3). The resulting list of preferentially expressed miRNAs was filtered in the following manner: 1) probeset data was collected when 6 data points were nontrivial - parental and IM3 subpopulations of all 3 human lines each produced adequate RNA hybridization for data above background, 2) the resulting fold change data was sorted according to highest observed fold-change, 3) the top 38 miRNAs, all with at least one fold change > 2, were analyzed looking for the direction of change between parental lines and IM-3 subpopulations to be similar between U87, U251, and U373 data. That is, we were interested in miRNAs that either moved uniformly up or down in invasive subpopulations. We found that miR-143 and -145 were over-expressed in all three IM3 cell lines compared to their parental counterparts (Figure 3B, red arrow). The expression level of miR-143 in IM3 cells is 2.5, 1.5 and 7.5 times higher than that of parental cells, while miR-145 was also overexpressed by 2, 1.2 and 4 fold (U87, U251 and U373 respectively) (Figure 3B). The parallel movement in expression between these molecules across cell lines was striking (Figure 3A vs. 3B). We also performed qRT-PCR for quantitation and validation of microarray results. As expected, the expression of these miRNAs differed significantly between parental and IM-3 cells: ~3 fold change of both miR-143 and -145 in U87, ~ 2 fold change of both miR-143 and -145 in U251, ~4 fold and ~3 fold change of miR-143 and -145 respectively in U373.

### The invasion of human GBM cells was down-regulated after treatment with antisense miR-143 and -145

**Figure 4:**
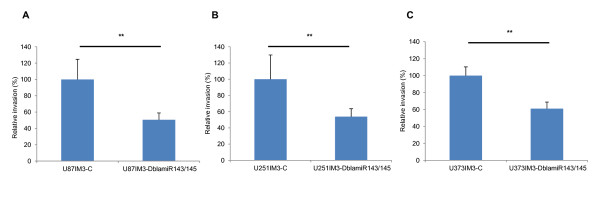
**Invasion assay with double antisense microRNA (DblamiR) treated IM3 cells**. U87 (A), U251 (B), and U373 (C) IM-3 cells were transfected with anti-miR-143 and anti-miR-145 (50 nM each) or with scrambled anti-miR (50 nM) as a control prior to running triplicate invasion assays. Data are represented as mean percentage of control treated cells ± SD. P: IM3: IM3 cells. C: scramble sequence miRNA control; amiR, antisense micro RNA; **, p < 0.01.

To confirm the role of miR-143 and -145 in enhanced invasiveness of IM3 cells, we tested the efficacy of combinatorial transfection of antisense LNA probes targeting human miR-143 and -145 (antagoMIR^® ^Exiqon) compared to untargeted/scrambled LNA probes. Double-treatment with antisense probes against miR-143 and -145 caused a decrease in invasion within the IM3 subpopulations (Figure 4A-C). The anti-invasive effect of these antimiRs is similar across all three human glioma cell lines: reducing invasion counts by 40 to 50% (Figure 4A-C). There was, however, a less predictable effect of treatment with either antimiR-143 or -145 alone (data not shown). These results suggest that double treatment with antimiR-143 and -145 has a synergistic effect in limiting the invasion of glioblastoma cells.

### Human glioblastoma expresses miR-143 and miR-145 in typical areas of invasion

In-situ hybridization confirmed the expression of miR-143 and miR-145 in human samples of glioblastoma. In freshly resected tumor, the expression appears prominently in the perivascular space, while tumors xenografted orthotopically into nude mice express these molecules near the tumor-brain interface 5.

## Discussion

In this study, we test the hypothesis that microRNAs are indeed key regulators of the invasive phenotype of glioblastoma. We propose that two specific micro-RNAs, miR-143 and miR-145, expressed from the same genetic locus [9], act in concert to promote glioma invasion. In contrast, miR-143 and miR-145 have been described by others as a tumor suppressor molecules in most non-neural human cancer cell lines tested [10,11]. Fewer reports suggest a tumor type-specific difference in their effect, and an oncogenic role in certain settings [12]. Finally, Cordes et al. suggest a role for miR-143/145 in promoting the differentiation of neural crest cells into vascular smooth muscle [9]. Future investigations will help define whether there is a causal connection between miR-143 and miR-145 promoting invasion and decreasing tumor growth or, alternatively, promoting more mesenchymal behavior.

### Serial selected invasive cell line (IM3)

The process of glioblastoma invasion into surrounding parenchyma is complex - involving cell attachment, cytoskeletal remodeling, membrane deformation, extracellular matrix proteolysis, detachment, and altered metabolic demands [13-15]. One model for examining the myriad of genetic and epigenetic alterations necessary for invasion was created in our laboratory via serial selection through Matrigel^® ^in a modified Boyden chamber [16]. Invasion towards a serum gradient, trypsinization, regrowth, and further iterative invasion proved to be a reproducible method for selecting invasive glioma cells. Further, the invasive phenotype of selected cells has remained constant through multiple passages (up to 20) and through freeze-thaw cycles (up to 3 tested). Even in the C6 rat glioma cell line, the most invasive of the four lines tested in these experiments, we were able to create a phenotypically distinct subpopulation.

### Overexpression of miR-143 and miR-145 in glioblastoma cells

From our collection of cell lines and their more invasive sub-populations, we extracted RNA and generated miRNA expression data by hybridization to a popular microarray platform [17]. Accordingly, we were interested in miRNAs whose expression was increased in all the invasive subpopulations compared to their parental lines, or those miRNAs whose expression was uniformly decreased in invasive cells. Data used to make this decision was derived from the three human glioma lines (U87, U373, U251), all using the same human-specific Exiqon expression array platform. Although we had no *a priori *knowledge of the genetic loci involved, we noticed a significant parallel in the pattern of upregulation of miR-143 and miR-145 across the three human lines (Figure 3B). Investigation of the chromosomal location of these miRNAs confirmed a reasonable explanation for the parallel expression - they are encoded in the same transcript [9,18].

### Expression of miR-143 and miR-145 in resected human glioblastoma samples

**Figure 5:**
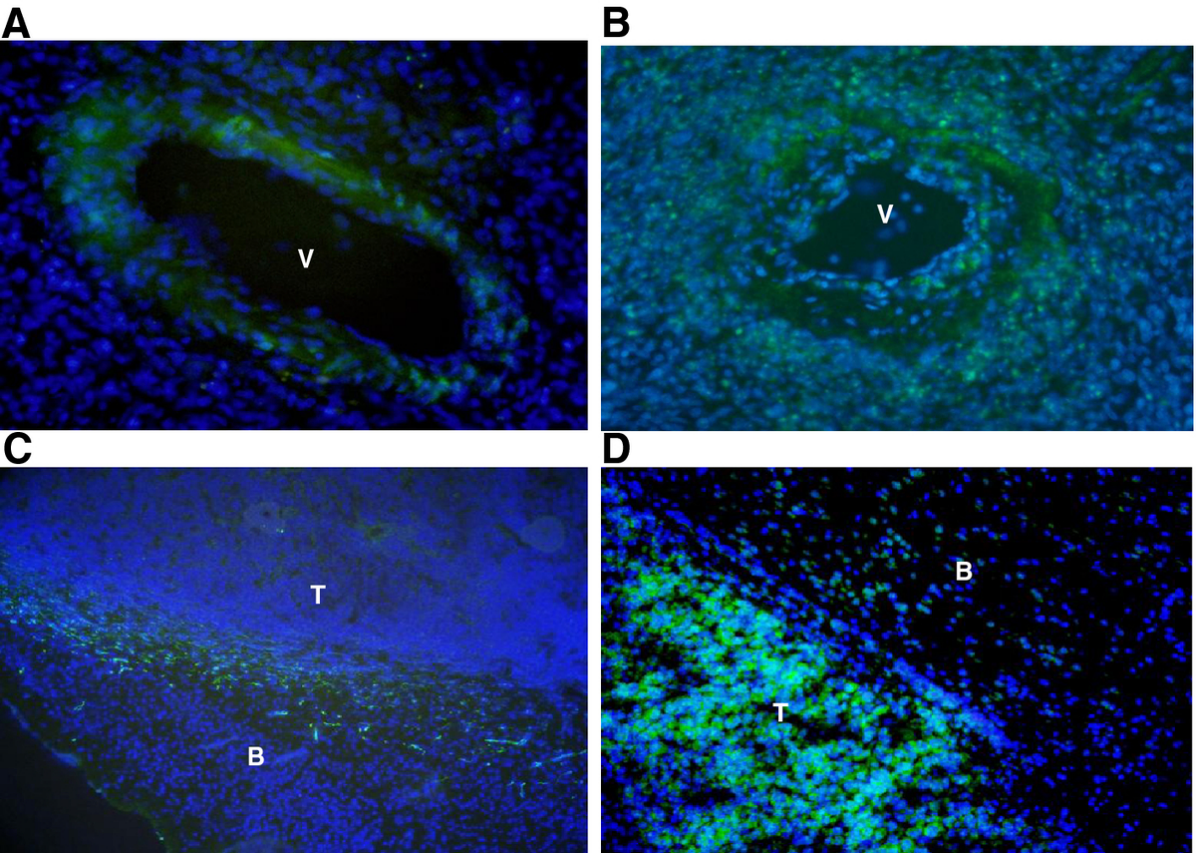
**Expression of miR-143 and -145 in human glioblastoma by miRNA probe *In situ *hybridization**. miRNA probe (FITC labeled miR-143 or -145) staining demonstrates the expression of miR-143 (A) and -145 (B) in frozen tissue section of human glioblastoma (V: blood vessel, patient sample) and the expression of miR-143 (C) and -145 (D) in human glioblastoma xenografted in frozen mouse brain section (T: tumor region; B: normal brain region).

Owing to its unique matrix composition, the use of Matrigel^® ^for selection of invasive glioma cells may bias our results towards identification of mediators of motility along basement membranes [19]. Yet, the propensity of glioblastoma to invade into these spaces: the perivascular Virchow-Robin space and the subpial plane, has been recognized since Scherer published landmark papers [20]. In our hands, *in situ *hybridization confirmed the expression of miR-143 and miR-145 along the perivascular space in frozen samples of resected human glioblastoma (Figure 5A-D). The downstream effects of the expressing the miR-143/-145 locus may allow for enhanced mobility along the unique extracellular matrix outside cerebral vasculature. In xenograft samples of glioblastoma, grown in the brains of nude mice, we found enhanced expression of miR-143 and miR-145 on either side of the tumor-host brain interface (Figure 5A, B). Although a typical basement membrane matrix has not been described at the interface between xenograft tumor and host brain, reactive astrocytes (fibronectin), and the invading tumor itself (vitronectin, proteoglycans) can both secrete ECM components shared by the Matrigel^® ^substrate [21,22]. Our selection of cells capable of invading through Matrigel^® ^may have identified those with cellular machinery allowing growth and invasion along the Virchow-Robin space and the brain-tumor interface. *In situ *regional expression of our target microRNAs in tumor samples illustrates, once again, the regional heterogeneity of glioblastoma, and supports our hypothesis about their role in mediating invasion.

### Synergistic anti-invasive effect of knocking down both of miR-143 and miR-145

In a series of transient transfection experiments, we have shown that antisense microRNAs treatment knocking down miR-143 and miR -145, in combination, abrogates the invasive phenotype of each of our human glioma cell lines (Figure 4A-C). Interestingly, in the U87 cell line, miR-145 knockdown alone was sufficient to produce a reduction in invasive phenotype (Data not shown), while in other cell lines, the combined treatment was needed to see the same effect. This finding underscores the necessity to characterize the expression of possible downstream targets across multiple human glioblastoma lines and primary cultures, and to find those characteristic differences between lines that account for the inconsistencies. More importantly, this points towards a molecular synergy that is not yet defined.

### Potential targets of miR-143/miR-145 and future directions

In an effort to characterize the important downstream targets of miR-143 and miR-145, we utilized three algorithms to score the likelihood of each to hybridize to, and affect translation of, particular genes. The on-line search algorithms TargetScan, PicTar, and MicroCosm were all used to stratify targets. By filtering the resulting lists for those targets that were identified by multiple search algorithms, we found a number of potential miR-145 targets (especially srGAP1) in the Slit/Robo pathway, which was recently recognized as an inhibitor of brain tumor cellular migration and invasion [23,24]. In western-blot experiments, we recognized a decreased expression of srGAP1 in the invasive subpopulation of U87 cells. Further definition of the role of srGAP1 activity, along with clearer understanding of other downstream mediators of both miR-143 and miR-145 is necessary.

The relationship between miR-143/miR-145 expression and other known mediators of glioma invasion must be defined. Very recently, Yan et al. described the likely micro-RNA regulators of MMP-9 expression - a molecule with known importance in survival and invasion of glioblastoma [25]. According to their results, 14 miRNAs (including miR-143, miR-210 and miR-214) positively regulate the overexpression of MMP-9 in glioma cell lines we tested (U87 and U251). Their results support our contention that miR-143 mediates invasion. Further, on review of our expression data, we also find miR-210 and miR-214 are upregulated in our IM-3 lines compared to their parental controls. Further studies are needed to identify the correlation between the expression of microRNAs and matrix metalloproteases in glioblastoma.

The identification of miR-143 and miR-145 as positive regulators of glioblastoma invasion is novel. In fact, most authors have described these molecules as tumor suppressors and mediators of differentiation into vascular smooth muscle [9,12,18]. A single report supports the role of miR-145 as an oncogene in metastatic colorectal cancer cells [12,26]. However, hypotheses based on data generated from malignancies outside the central nervous system often prove unsupported in glioblastoma. The behavior of this tumor is unique - it is markedly invasive in the host organ yet metastases are almost non-existent. The tumor grows quickly, but invasive cells are often slower-growing [27]. Emerging data from our laboratory and those of other investigators support a role for the miR-143/145 locus in promoting glioma invasion.

## Conclusions

The micro-RNA mediators of glioblastoma invasion are incompletely defined. We present, in this publication, a method for creating stable and invasive subcultures of common glioma cell lines, and we use them to define the micro-RNA regulators of invasion. Two molecules of interest, miR-143 and miR-145, are likely key pro-invasive mediators, and our data correlate well with those emerging from other investigators. Understanding the unique pathophysiology of glioblastoma invasion will help direct future drug design and therapies aimed at prolonging meaningful quality of life.

## Competing interests

The authors declare that they have no competing interests.

## Authors' contributions

LGT performed the initial project design, the experimental design, and edited the manuscript and data analysis. SWK participated in the experiment, performed data analysis, and wrote the initial manuscript. GM was a key developer of the experimental approach, KS performed and interpreted experiments, and MR designed the methodology for miRNA target analysis. All authors read and approved the final manuscript.

## Pre-publication history

The pre-publication history for this paper can be accessed here:

http://www.biomedcentral.com/1471-2407/12/143/prepub

## Supplementary Material

Additional file 1 **The invasion and attachment raw plots**. Invasion assay (A) and attachment assay (B) data from glioblastoma cell lines: U87, U251, U373, C6. (blue rectangles) Parental (red rectangles) IM3. Click here for file
