# Supplementary material for: Serial selection for invasiveness increases expression of miR-143/miR-145 in glioblastoma cell lines
Source: BMC Cancer. 2012 Apr 10;12:143. doi: 10.1186/1471-2407-12-143 (PMC3378456; doi:10.1186/1471-2407-12-143)
Supplement: Additional file 1 — The invasion and attachment raw plots. Invasion assay (A) and attachment assay (B) data from glioblastoma cell lines: U87, U251, U373, C6. (blue rectangles) Parental (red rectangles) IM3. [file 1471-2407-12-143-S1.PDF]

A

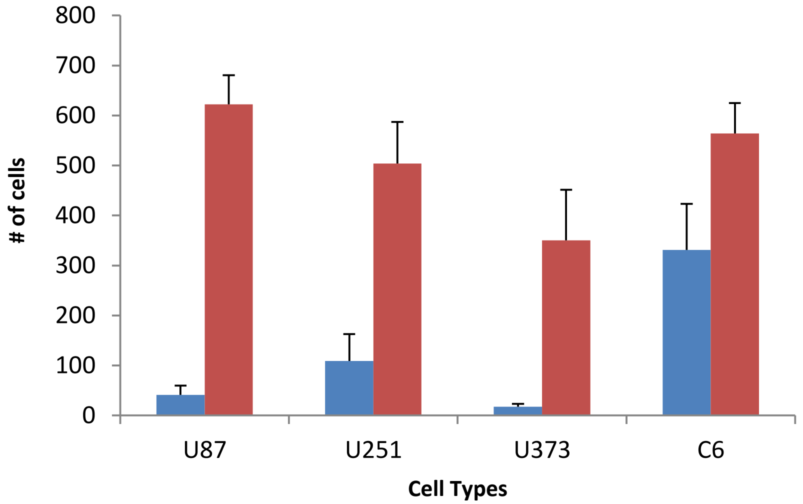
800
700
600
500
400
300
200
100
0
# of cells
U87
U251
U373
C6
Cell Types

B

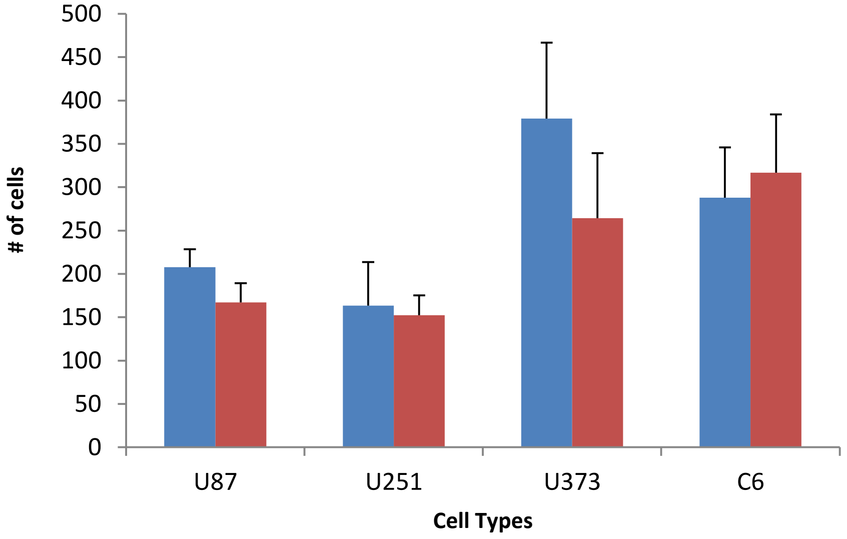
500
450
400
350
300
250
200
150
100
50
0
# of cells
U87
U251
U373
C6
Cell Types

Additional File 1

The invasion and attachment raw plots.
Invasion assay (A) and attachment assay (B) of glioblastoma cell lines: U87, U251, U373, C6.
Parental IM3
